# Supplementary material for: Chronic microfiber exposure in adult Japanese medaka (Oryzias latipes)
Source: PLoS One. 2020 Mar 9;15(3):e0229962. doi: 10.1371/journal.pone.0229962 (PMC7062270; doi:10.1371/journal.pone.0229962)
Supplement: S1 Fig — Different concentrations of PP (A) and PES (C) MFs dispersed in 10 mL 70% ethanol. Standard curves of PP (B) and PES (D) MFs. (DOCX) [file pone.0229962.s001.docx]

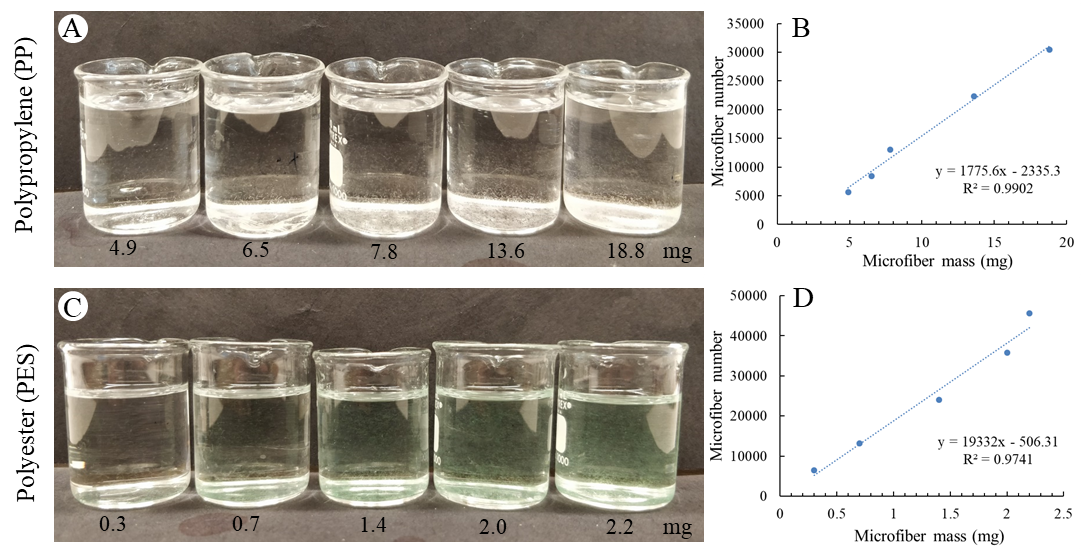


**S1 Fig.** **Standard Curves.** Different concentrations of PP (A) and PES (C) MFs dispersed in 10 mL 70% ethanol. Standard curves of PP (B) and PES (D) MFs.
