# Supplementary material for: Chronic microfiber exposure in adult Japanese medaka (Oryzias latipes)
Source: PLoS One. 2020 Mar 9;15(3):e0229962. doi: 10.1371/journal.pone.0229962 (PMC7062270; doi:10.1371/journal.pone.0229962)
Supplement: S2 Fig — Body weights of female (A) and male (B) medaka before (light grey bars) and after exposure (dark grey bars). Medaka were exposed to 0 (Control), PP, or PES MFs for 21 days (n = 18). Data are presented as means ±SD. Mann-Whitney U-test and Wilcoxon tests were used to determine the differences in the body weight of medaka among different treatment groups and between before and after exposure, respectively. # p < 0.05, ## p < 0.01. (DOCX) [file pone.0229962.s002.docx]

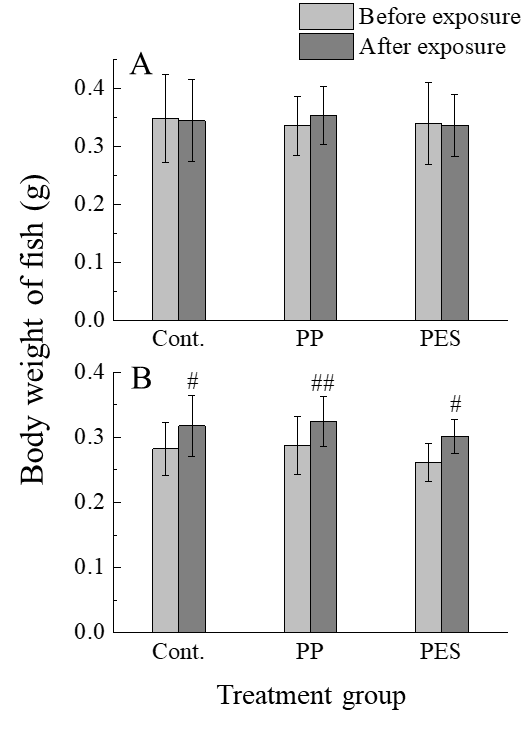


**S2 Fig. Body weights of female (A) and male (B) medaka before (light grey bars) and after exposure (dark grey bars).** Medaka were exposed to 0 (Control), PP, or PES MFs for 21 days (n=18). Data are presented as means ±SD. Mann-Whitney *U*-test and Wilcoxon tests were used to determine the differences in the body weight of medaka among different treatment groups and between before and after exposure, respectively. # p < 0.05, ## p < 0.01.
