# Supplementary material for: Chronic microfiber exposure in adult Japanese medaka (Oryzias latipes)
Source: PLoS One. 2020 Mar 9;15(3):e0229962. doi: 10.1371/journal.pone.0229962 (PMC7062270; doi:10.1371/journal.pone.0229962)
Supplement: S3 Fig — Survival rate (A-C) and hatching percent (D-F) of embryos collected at day 7 (A, D), 14 (B, E) and 21 (C, F). Data are presented as means, n = 5–9 tanks. PP, Polypropylene MFs; PES, Polyester MFs. (DOCX) [file pone.0229962.s003.docx]

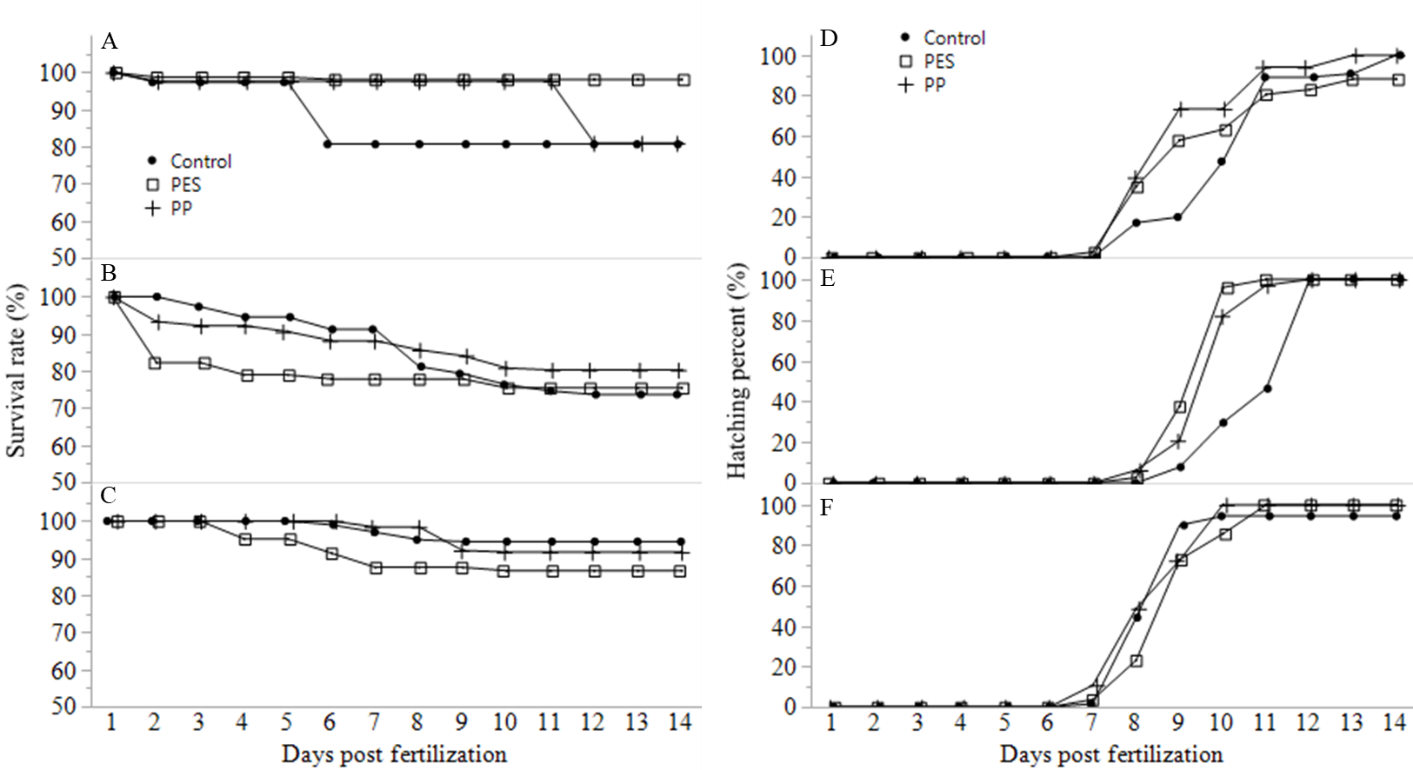


**S3 Fig.** **Embryo survival and hatching.** Survival rate (A-C) and hatching percent (D-F) of embryos collected at day 7 (A, D), 14 (B, E) and 21 (C, F). Data are presented as means, n=5-9 pairs. PP, Polypropylene MFs; PES, Polyester MFs.
