# Supplementary material for: Chronic microfiber exposure in adult Japanese medaka (Oryzias latipes)
Source: PLoS One. 2020 Mar 9;15(3):e0229962. doi: 10.1371/journal.pone.0229962 (PMC7062270; doi:10.1371/journal.pone.0229962)
Supplement: S4 Fig — Malformation rates of larvae at 14 days post fertilization (dpf) from control, PP, and polyester PES MFs for 7 (A), 14 (B) and 21 (C) days. Data are presented as medians, n = 5–9 tanks. (DOCX) [file pone.0229962.s004.docx]

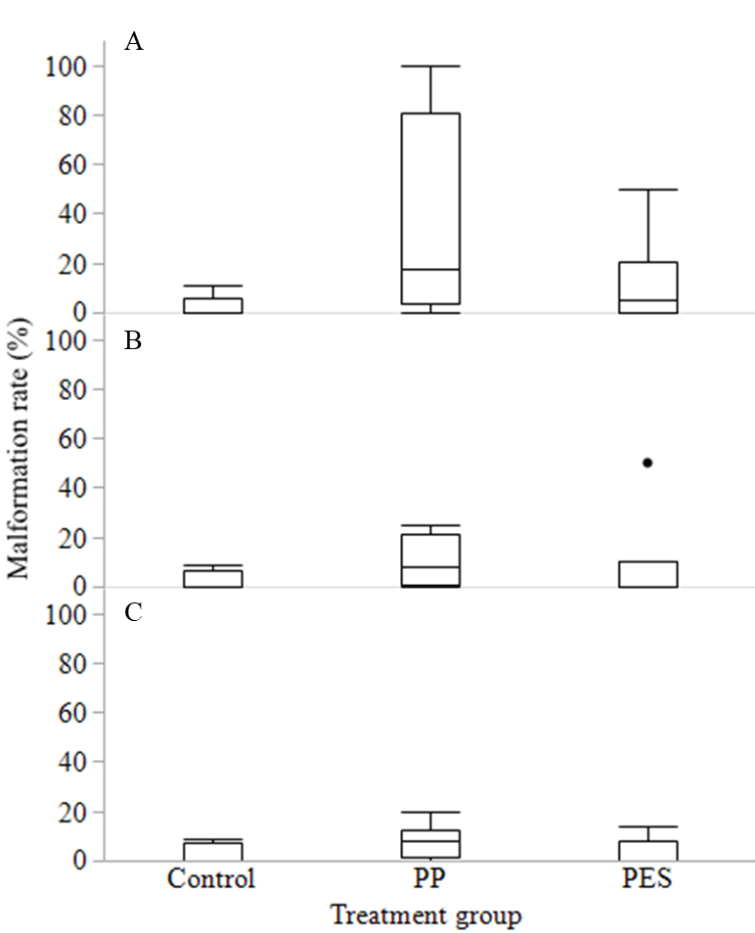


**S4 Fig. Malformation rate of larvae.** Malformation rate of larvae at 14 days post fertilization (dpf) from control, PP, and polyester PES MFs for 7 (A), 14 (B) and 21 (C) days. Data are presented as medians, n=5-9 pairs.
