# Supplementary material for: Chronic microfiber exposure in adult Japanese medaka (Oryzias latipes)
Source: PLoS One. 2020 Mar 9;15(3):e0229962. doi: 10.1371/journal.pone.0229962 (PMC7062270; doi:10.1371/journal.pone.0229962)
Supplement: S5 Fig — Body length at 14 days post fertilization (dpf) larvae exposed to MFs for 14 and 21 days. Data are presented as medians ± SD, n = 5–9 tanks. (DOCX) [file pone.0229962.s005.docx]

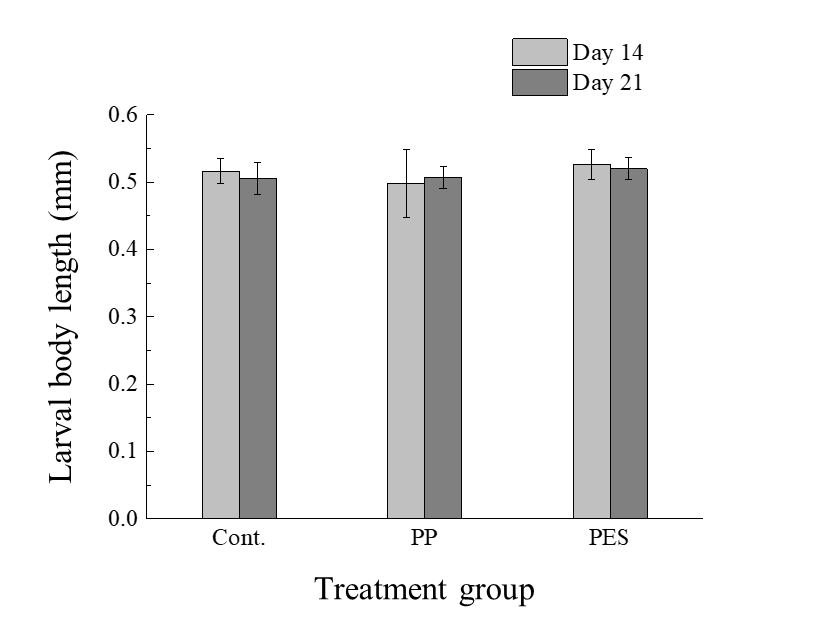


**S5 Fig. Body lengths of larvae.** Body length at 14 days post fertilization (dpf) larvae exposed to MFs for 14 and 21 days. Data are presented as medians ± SD, n=5-9 pairs.
