# Supplementary material for: Chronic microfiber exposure in adult Japanese medaka (Oryzias latipes)
Source: PLoS One. 2020 Mar 9;15(3):e0229962. doi: 10.1371/journal.pone.0229962 (PMC7062270; doi:10.1371/journal.pone.0229962)
Supplement: S6 Fig — H&E stained sections of mouth (A), buccal cavity (B) and pharynx near teeth (C) from control fish. H&E stained sections of mouth (D), buccal cavity with high magnification inset of MF (E), pharynx near teeth with high magnification inset of MF (F), gill filaments with high magnification inset of MF in direct contact with outgrowths on secondary lamella (G) and gut (H) from PES-exposed fish. AB-PAS stained sections of gut (I) from PP-exposed fish with wall of gut at bottom of field and gut lumen occupying middle to upper portions of field; PP MFs in negatively stained, clear spaces signifying former presence of MFs. Low and high magnification images with black arrows indicate MFs. (DOCX) [file pone.0229962.s006.docx]

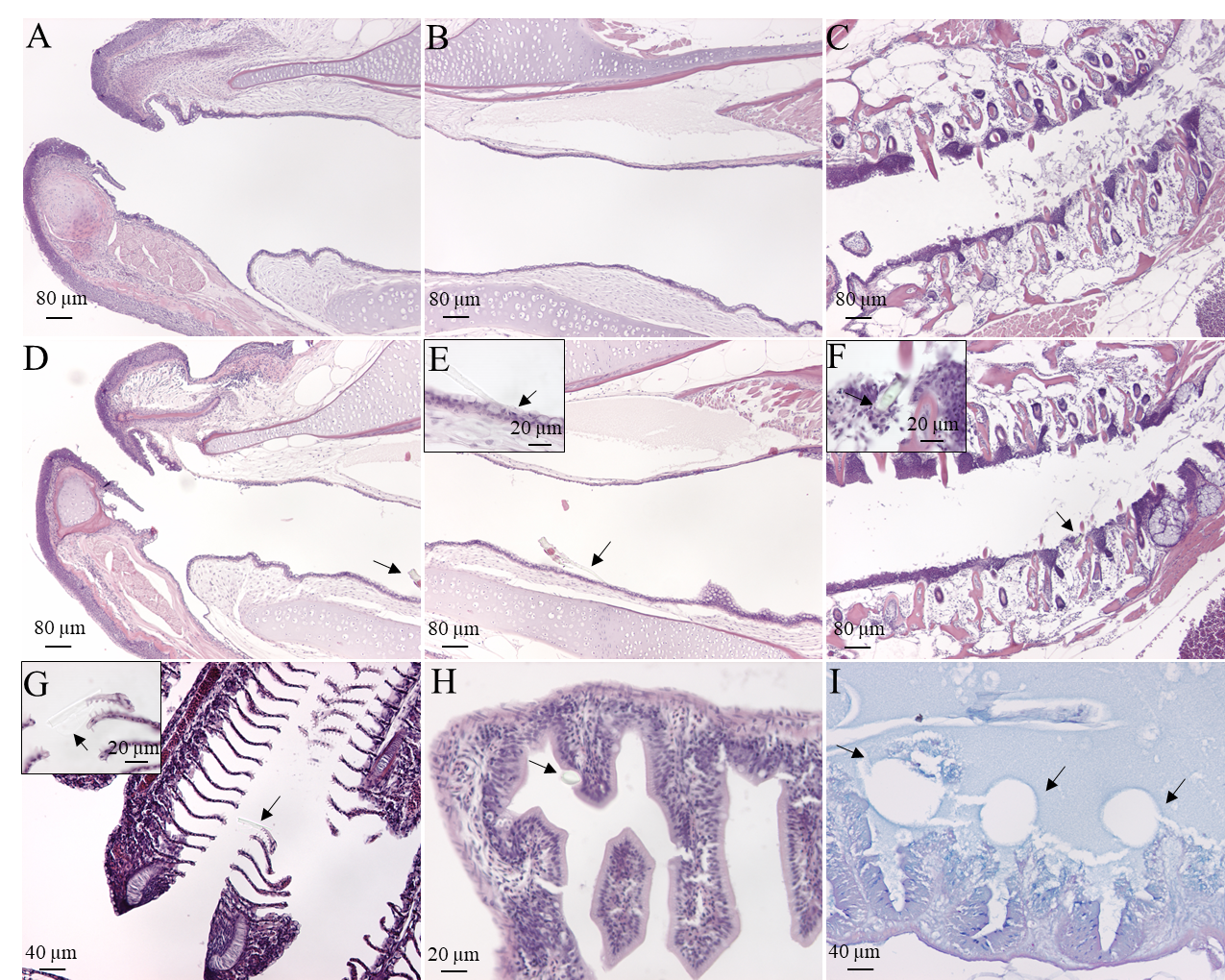


**S6 Fig. Histological micrographs of MF distribution in medaka.** Histological micrographs of MF distribution in medaka. H&E stained sections of mouth (A), buccal cavity (B) and pharynx near teeth (C) from control fish. H&E stained sections of mouth (D), buccal cavity with high magnification inset of MF (E), pharynx near teeth with high magnification inset of MF (F), gill filaments with high magnification inset of MF in direct contact with outgrowths on secondary lamella (G) and gut (H) from PES-exposed fish. AB-PAS stained sections of gut (I) from PP-exposed fish with wall of gut at bottom of field and gut lumen occupying middle to upper portions of field; PP MFs in negatively stained, clear spaces signifying former presence of MFs. Low and high magnification images with black arrows indicate MFs.
