# Supplementary material for: Chronic microfiber exposure in adult Japanese medaka (Oryzias latipes)
Source: PLoS One. 2020 Mar 9;15(3):e0229962. doi: 10.1371/journal.pone.0229962 (PMC7062270; doi:10.1371/journal.pone.0229962)
Supplement: S7 Fig — AB-PAS stained histological sections in foregut after 21-day exposure to 0 (control; A-B), PES (C-D), or PP (E-F) MFs. (B, D, F) The higher magnification views of areas in the foregut indicated by squares in A, C, and D. Black arrows indicate goblet cells. (DOCX) [file pone.0229962.s007.docx]

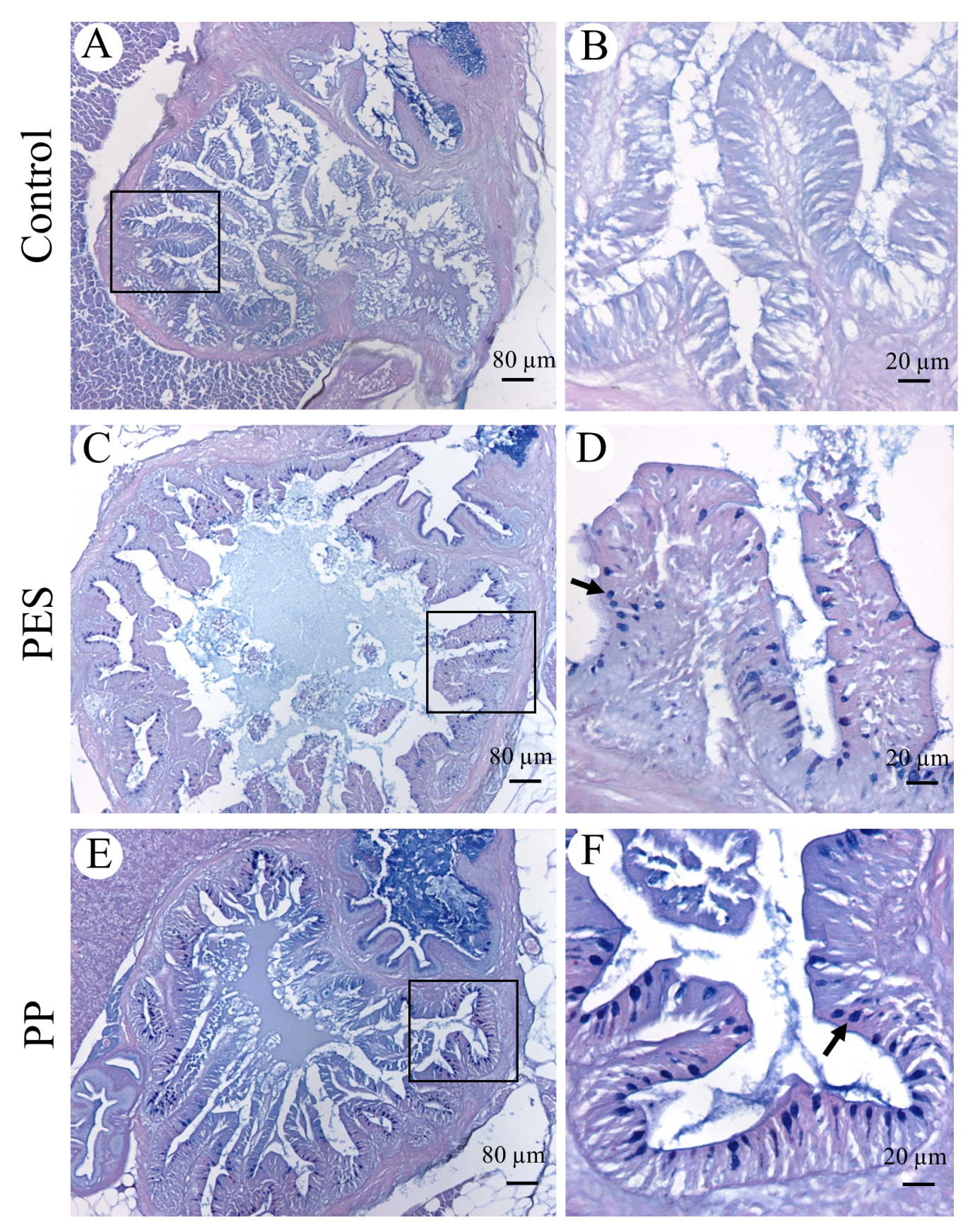


**S7 Fig.** **AB-PAS stained histological sections in foregut** after 21-day exposure to 0 (control; A-B), PES (C-D), or PP (E-F) MFs. (B, D, F) The higher magnification views of areas in the foregut indicated by squares in A, C, and D. Black arrows indicate goblet cells.
