# Supplementary material for: Chronic microfiber exposure in adult Japanese medaka (Oryzias latipes)
Source: PLoS One. 2020 Mar 9;15(3):e0229962. doi: 10.1371/journal.pone.0229962 (PMC7062270; doi:10.1371/journal.pone.0229962)
Supplement: S1 Table — Mass concentration (mg/L) of PP and PES MFs at the test concentration of 10,000 microfibers/L used for this study. (DOCX) [file pone.0229962.s008.docx]

**S1 Table. Test Concentrations.** Mass concentration (mg/L) of PP and PES MFs at the test concentration of 10,000 microfibers/L used for this study.

| Microfiber | Comparison of concentrations | |
| --- | --- | --- |
|  | microfibers/L | mg/L |
| Polypropylene (PP) | 10,000 | 6.29 |
| Polyester (PES) | 10,000 | 0.53 |
