# Supplementary material for: Chronic microfiber exposure in adult Japanese medaka (Oryzias latipes)
Source: PLoS One. 2020 Mar 9;15(3):e0229962. doi: 10.1371/journal.pone.0229962 (PMC7062270; doi:10.1371/journal.pone.0229962)
Supplement: S2 Table — (DOCX) [file pone.0229962.s009.docx]

**S2 Table.** **Measurements of adult fish exposed to MFs for 21 days.**

| Treatment group | Control | | Polypropylene (PP) | | Polyester (PES) | |
| --- | --- | --- | --- | --- | --- | --- |
| Sex | Females | Males | Females | Males | Females | Males |
| Standard length (mm) | 28.4 ± 2.4 ^a^ | 28.1 ± 1.5 | 27.8 ± 1.9 | 27.9 ± 1.2 | 28.1 ± 1.9 | 27.3 ± 1.1 |
| Girth (mm) | 7.0 ± 0.7 | 6.8 ± 0.5 | 6.9 ± 0.6 | 6.9 ± 0.6 | 6.9 ± 0.5 | 6.5 ± 0.4 |
| Gill weight (mg) | 7.7 ± 1.9 | 7.8 ± 2.2 | 7.4 ± 1.7 | 7.5 ± 1.4 | 6.1 ± 0.7 | 8.6 ± 1.6 |
| Gut weight (mg) | 19.5 ± 7.2 | 14.3 ± 6.4 | 16.5 ± 4.4 | 11.3 ± 2.6 | 18.1 ± 1.9 | 10.6 ± 3.0 |
| Liver weight (mg) | 14.3± 7.1 | 8.2± 4.0 | 16.5± 4.4 | 7.6± 2.1 | 11.4± 2.0 | 7.3± 1.5 |
| Gonad weight (mg) | 21.3 ± 7.1 | 3.8 ± 1.6 | 21.5 ± 2.6 | 4.1± 1.7 | 21.1 ± 4.1 | 2.7 ± 1.2 |
| *K* | 1.5 ± 0.1 | 1.4 ± 0.1 | 1.5 ± 0.2 | 1.5 ± 0.2 | 1.7 ± 0.2 | 1.5 ± 0.2 |
| HSI (%) | 4.7 ± 2.3 | 2.6 ± 1.1 | 3.4 ± 1.3 | 2.3 ± 0.7 | 3.7 ± 0.8 | 2.4 ± 0.6 |
| GSI (%) | 7.0 ± 2.3 | 1.2 ± 0.3 | 7.5 ± 0.9 | 1.2 ± 0.4 | 6.8 ± 1.0 | 0.9 ± 0.4 |

^a^ Values represent mean ± SD. *K*, coefficient of condition (n=18); HSI (%), hepatosomatic index (n=6 females, 6 males); GSI (%), gonadosomatic index (n=6 females, 6 males). Mann-Whitney *U*-test was used to determine differences among different treatment groups.
